# A population-based projection of psoriatic arthritis in Germany until 2050: analysis of national statutory health insurance data of 65 million German population

**DOI:** 10.1007/s00296-023-05422-2

**Published:** 2023-08-19

**Authors:** Jiancong Wang, Sabrina Tulka, Stephanie Knippschild, Matthias Schneider, Jörg H. W. Distler, Xenofon Baraliakos, Ralph Brinks, Philipp Sewerin

**Affiliations:** 1 Institute of Biometry and Epidemiology, The German Diabetes Center, Heinrich Heine University Düsseldorf, 40225 Düsseldorf, Germany; 2 Chair for Medical Biometry and Epidemiology, University of Witten/Herdecke, 58448 Witten, Germany; 3 Hiller Research Center, University Hospital Düsseldorf, Medical Faculty of Heinrich Heine University, 40225 Düsseldorf, Germany; 4 Clinic for Rheumatology, University Hospital Düsseldorf, Medical Faculty of Heinrich Heine University, 40225 Düsseldorf, Germany; 5 Ruhr-Universität Bochum, Rheumazentrum Ruhrgebiet, 44649 Herne, Germany

**Keywords:** Psoriatic arthritis, Population-based projection, Prevalence, Incidence, Illness–death model, Germany

## Abstract

The population-based prevalence of psoriatic arthritis (PsA) is still unclear and not well described globally. The aim of this study was to conduct a population-based prevalence projection and provide long-term future estimations of PsA patients in Germany until 2050, using the illness–death model and based on historical data. We analyzed the national statutory health insurance data of 65 million population in the German Institute for Medical Documentation and Information between January 2009 and December 2012. We constructed an estimation of the PsA burden among the German population using the relevant epidemiological parameters to project the numbers of patients with PsA in Germany until 2050 under five possible scenarios by varying the incidence and mortality. The overall conservatively estimated prevalence of PsA in Germany in 2019 was 0.31% (95% CI 0.28–0.36%). Women contribute a higher prevalence than men in all five scenarios. In the assumed scenarios with increased incidence, the prevalence of PsA at 60 years of age could rise from 1% in 2019 to more than 3% in 2050 for both genders, with the increase particularly pronounced for women, reaching around 3.5%. However, in the assumed scenarios with decreasing incidence, the prevalence curve may flatten and begin a decreasing trend from 2035 to 2050 for both genders, achieving a prevalence of less than 1% in 2050. Our research is to generate assumed population-based data on PsA in Germany that can serve as a reference for public health stakeholders to prepare an optional intervention. We would expect worryingly high numbers in the coming decades if preventive strategies are not implemented. In the long term, it will be necessary to implement preventive strategies to identify predictors and treat psoriasis symptoms early in order to delay or even prevent the transition of psoriasis to PsA.

**Supplementary Information:**

The online version contains supplementary material available at 10.1007/s00296-023-05422-2.

## Introduction

Psoriatic arthritis (PsA) is an inflammatory disorder that most often occurs after skin psoriasis and typically involves the peripheral joints, entheses, or axis, which can potentially lead to chronic pain and impaired functioning [1–3]. For patients, there is a risk of permanent damage, deformity, and disability, which can all impact health-related quality of life and disability-adjusted life years and are each associated with increased social healthcare expenses [3, 4]. However, PsA can be diagnosed and treated early, thus delaying the progression of the disease, if awareness is raised among those with risk factors for musculoskeletal involvement and if they are persuaded to seek early help from a rheumatologist [5].

PsA is a chronic inflammatory disease that occurs more rarely than other non-communicable diseases, and the global epidemiological burden varies by location, with more data available in high-income countries [6, 7]. Scotti et al. in a systematic review, reported that the prevalence of PsA in the general population was 0.13%, with an incidence of 83 per 100,000 person-years [7]. In Germany, Maximilian et al. estimated the prevalence of PsA, using the German health insurance database, to be 0.29% in the adult population in 2010, with the highest prevalence of 0.50% among those in their 50 s [8]. In Norway, Hoff et al. estimated the prevalence in the adult population to be between 0.67% and 0.70%, with an incidence of 41.3 per 100,000 person-years during 2000–2008 [9]. In the USA, Karmacharya et al. estimated the prevalence of PsA in the general population at 0.18%, with a relatively low incidence of 8.5 per 100,000 population in 2000–2017 [10].

The global population-based prevalence of PsA is still unclear and not well described [11]. Only a few countries, such as Denmark, Canada, and the USA, have the capability to organize and publish their PsA population-based surveillance data using a national medical registration system [10, 12, 13]. The global rheumatology literature also lacks data to describe the developments of PsA over certain time periods [11]. Given the limits of existing medical sources, health scientists are encouraged to use a projection model to estimate or forecast future situations and possible trends and developments of PsA based on historical data [14–16]. Existing research offers examples of successful projections and estimations of other non-communicable diseases using the illness–death model [14–16], but there are so far no studies in the global rheumatology literature that have applied such mathematical model to project and estimate the future burden of PsA. At the national level, projection estimations could help public health stakeholders to better prepare pharmaceutical interventions and medical resource allocation in order to improve the quality of life of patients with PsA [17].

In this analysis, we used population-based data of 65 million from the German Institute for Medical Documentation and Information (DIMDI) between January 2009 and December 2012 [18]. The specific aims were (i) to conduct a population-based prevalence projection and provide long-term future estimations of PsA patients in Germany until 2050, using the illness–death model and based on historical data; and (ii) using assumptions and references from the literatures, to compare estimations of PsA patient numbers between five different possible scenarios that reflect potential trends and developments of the disease.

## Methods

### Study population datasets

The data of 65 million for the study were extracted from the nationwide healthcare registry in Germany. For the PsA dataset, we used the population-based aggregate of patients diagnosed with PsA who were originally registered with the DIMDI [18]. The PsA diagnoses were made by physicians according to the 10th edition of the International Statistical Classification of Diseases and Related Health Problems (ICD-10) [19], and around 90% of the population in Germany is covered by statutory health insurance [20]. The PsA data were mainly structured as two aggregate datasets: the population-based prevalence and incidence rates of PsA, each of them with stratification into different age groups and genders between 2009 and 2012. Deike et al. presented the fundamental structure of the data, along with the age-specific and sex-specific prevalence and incidence of PsA [21]. While for the general population projections, we used projection datasets from the German Federal Statistical Office, which are constantly updated forecasts of population numbers in Germany until 2060 based on the changes in the birth rate, life expectancy, and migration [22].

### Study design and health outcome

The study design was an observational, population-based, prospective study that used a projection model to predict future trends of PsA based on the historical data. The concept of the study is an estimation of the PsA disease burden among the German population, incorporating existing epidemiological parameters (i.e., prevalence, incidence, general population mortality, and PsA mortality) into the illness–death model [15, 23]. The health outcome is a projection of the numbers of patients with PsA in Germany until 2050, based upon five possible scenarios that reflect trends and developments of the disease.

### Inclusion and exclusion criteria

We included all aggregated insurance data from 2009 to 2012 regarding the prevalence and incidence of PsA; no data were excluded from the study.

### Illness–death model for the projection of Psoriatic arthritis

#### Prevalence

We firstly applied a linear regression model with interaction effects [24]. This model was used to predict and iteratively interpolate the prevalence of PsA for each year from 2009 to 2012 and for every age from birth to 100 years old. We then derived the forecasted prevalence values until 2050 based on the extrapolation of these interpolated historical prevalence data [21]. To fit the linear model, we applied the natural logarithm function of the historical prevalence between 2009 and 2012, which was considered the dependent variable; and incorporated the parameters of the year, the cubic spline function of the age groups, and sex as independent variables (Supplementary Table 1) [21, 25].

#### Incidence

Methodologically similar to the prevalence estimates, we firstly applied a linear regression model to predict the incidence of PsA for each year from 2009 to 2011 and for every age from birth to 100 years old. We then derived the forecasted incidence values until 2050 from the extrapolation of these interpolated historical incidence data [21]. To fit the linear model, we applied the natural logarithm function of the historical incidence between 2009 and 2011, which was considered the dependent variable; and incorporated the cubic spline function of the age groups and sex as independent variables [21, 25]. For validation, we included the specific values of age and sex as independent variables in the incidence prediction function equation, which yielded similar results corresponding to the existing historical incidence values by age and sex.

#### General mortality rate ratio

The general population mortality rate ratio (MRR) calculation was based on concepts from the Strehler–Mildvan model [26], which incorporated gender, socioeconomic status, and access to healthcare in the context of modern health system development. The predicted annual values calculated from the global population MRR trends matched those of the general population, stratified by sex.

#### Mortality rate ratio for psoriatic arthritis

For the MRR of PsA, the primary data references came from a systematic literature search, which yielded a Danish nationwide cohort study that reported MRRs of PsA to all-cause mortality of 2.23 in the 18–50 age group, 1.87 in the 51–70 age group, and 1.43 in the over-70 age group for the interpolation function for all MRRs corresponding to every age from birth to 100 years old [12]. Given that Denmark shares a comparable socioeconomic and demographic status and lifestyle with Germany, it is reasonable for us to infer that the MRR of PsA in Denmark is most likely to be similar to that in Germany. Therefore, we consider it justifiable to use the Danish data as a reference for MRR of PsA in our study.

#### Illness–death model

The epidemiological parameters of PsA, including the predicted prevalence, incidence, general population MRR, and relative MRR for PsA, form the basis for the illness-death model (Supplementary Fig. 1). The advantage of this model captures disease progression and transitions between states and stages [23, 27]. We referred to Brinks et al.’s mathematical concepts for modeling non-communicable diseases, which are based methodologically on partial differential equations, incorporating the availability of epidemiological parameters [23]. The applicability of the model has been confirmed and it has demonstrated superiority over a simple projection-based prevalence approach [28]. Moreover, the model is versatile and can be used in various scenarios for estimating the burden of non-communicable diseases from an epidemiological perspective [23].

### Five scenarios for psoriatic arthritis trend projections

The nature of illness-death model suggests that the projections were highly responsive to variations in both incidence and mortality rates. We hypothesized about future trends in PsA and its disease progression, consulting credible sources and references, and devising five distinct scenarios that account for all possible developments between 2019 and 2050 (Supplementary Table 2A and B).

In the first scenario, as a conservative baseline estimate, we assumed that the incidence and mortality of PsA from 2019 onwards would remain constant and the same as at present. In the second scenario, we assumed that the mortality of PsA would decrease by 2% every year on average and that incidence would decrease by 2.5% every year, as similar declines have been seen in the other literature (Table 1) [29–31]. We assumed that the incidence and mortality of PsA would both decrease because of early diagnosis, active screening, and early referral to rheumatology clinics [3], and that patients would increasingly receive appropriate treatment with non-steroidal anti-inflammatory drugs and local glucocorticoid injections as initial therapy, as recommended by the European League Against Rheumatism [32].

**Table 1:** Summary of five different scenarios for the developments of psoriatic arthritis, based on information from published literature and outlined changes in incidence and mortality rates over the years—Population-Based Projection in Germany

Scenario	Incidence		Mortality	
	Literature	Change of rate by year	Literature	Change of rate by year
Scenario 1	N/A	Remain constant	N/A	Remain constant
Scenario 2	Guldberg-Møller et al. [29]	≈ 2.5% (↓)	Springate et al. [30]; Iskandar et al. [31]	≈ 2.0% (↓)
Scenario 3	Love et al. [33]; Green et al. [34]	≈ 2.5% (↑)	Chaudhary et al. [35]	≈ 2.0% (↑)
Scenario 4	Karmacharya et al. [10]; Eder et al. [13]	≈ 5.0% (↑)	N/A	Remain constant
Scenario 5	Hoff et al. [9]; Kerola et al. [38]	≈ 5.0% (↓)	N/A	Remain constant

The calculation for each scenario involving variations in incidence and mortality rates were outlined in Supplementary Table 2A and B

The literatures included to provide evidence for each scenario was obtained through systematic literature search, which took into account specific search terms for PubMed (via Medline) such as “mortality” [Title], “incidence” [Title], and “psoriatic arthritis” [Title]

*N/A* not applicable, (↓) decrease, (↑) increase

In the third scenario, we assumed that the mortality of PsA would increase by 2% every year on average and that the incidence would increase by 2.5% every year [33–35]. We assumed that, despite the development of PsA treatments, patients would be unable to receive proper treatment because of the costs of the drug, and the difficulties for accessibilities of the PsA patient care and management [36, 37]. It impacts the effectiveness of the therapies against the PsA (Table 1).

In the fourth scenario, we assumed that the mortality of PsA would remain constant, as medical advances and the introduction of effective therapies would at least keep the situation on the individual level from deteriorating [3], but that the incidence of PsA would increase by 5% every year [10, 13]. While in the fifth scenario, we again assumed mortality would be constant but that the incidence would decrease by 5% every year (Table 1) [9, 38].

### Statistical analysis

We used a Monte Carlo simulation to forecast the future prevalence of PsA from 2019 to 2050 for the German population at every age from birth to 100 years old, for both men and women. To solve the differential equation, we used a fourth-order Runge–Kutta method based on the epidemiological parameters from the illness–death model and the assumptions of the five different scenarios. Using projected PsA prevalence and the German population forecasts from the Federal Statistical Office, we projected the future numbers of PsA patients from 2019 to 2050 in the five different scenarios. All the analyses and graphs were produced using the statistical software R, version 4.2.1 (The R Foundation for Statistical Computing Platform). We also calculated the percentage changes in projected case numbers for the next three decades (using 2019 as the baseline year) in each of the five scenarios.

### Ethics and reporting

All the datasets in the current study were in an aggregated data format; written informed consent and ethical approval were therefore not required. This study has been approved and supported by the Hiller Research Center, University Hospital Düsseldorf, Medical Faculty of Heinrich Heine University. All authors had full access to the study dataset and take responsibility for data protection protocols. This study was reported in accordance with the REporting of studies Conducted using Observational Routinely-collected Data (RECORD) guideline [39].

## Results

### Projected prevalence of psoriatic arthritis

**Fig. 1:**
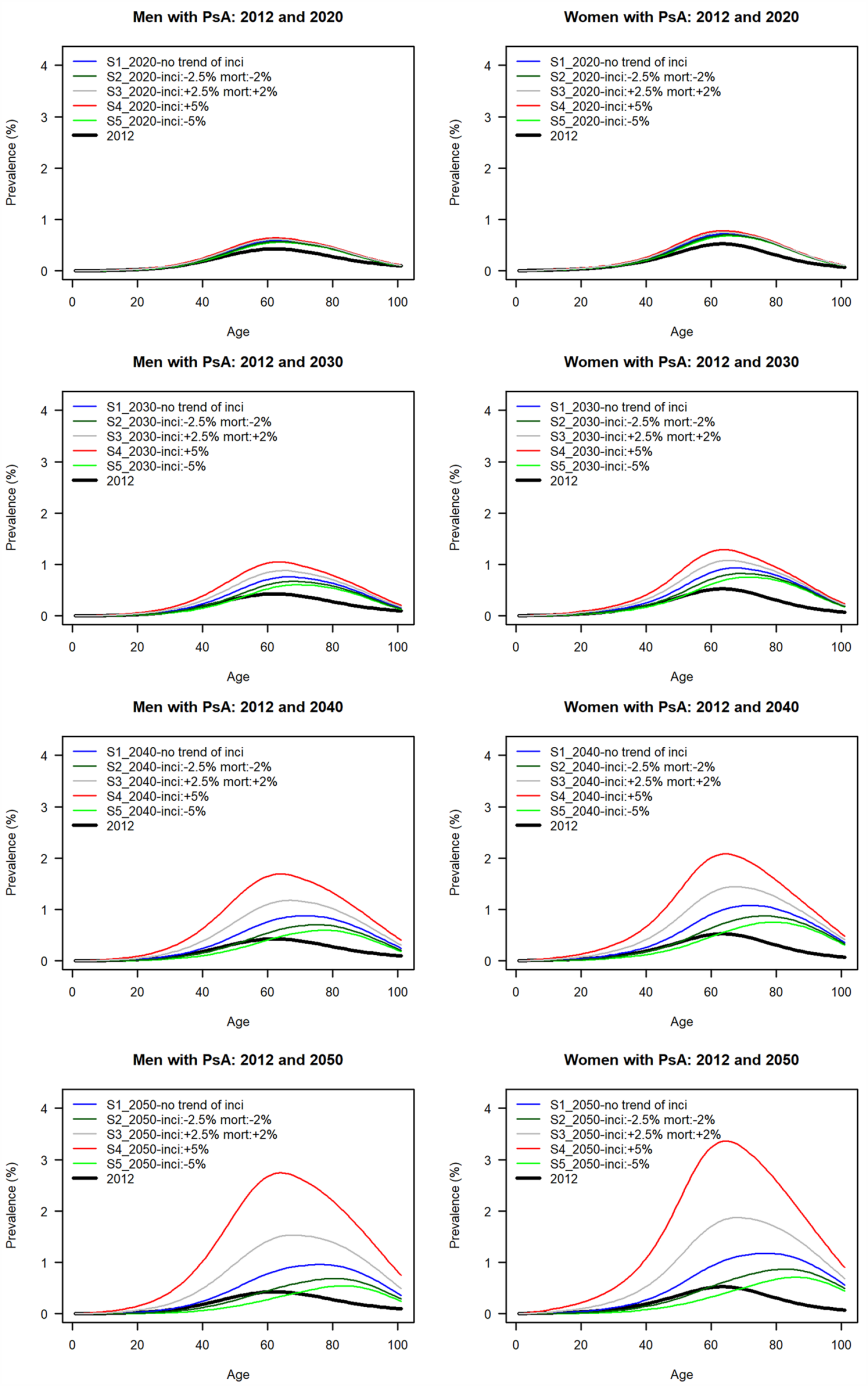
Projected age-specific prevalences of psoriatic arthritis for men and women in the five scenarios in 2020, 2030, 2040, and 2050—Population-Based Projection in Germany. The bold black lines represent the historical prevalences with age-specifics in 2012. Scenario 1: The incidence and mortality of psoriatic arthritis from 2019 onwards would remain constant and the same as at present; Scenario 2: The mortality of psoriatic arthritis would decrease by 2% every year on average and the incidence would decrease by 2.5% every year from 2019 onwards; Scenario 3: The mortality of psoriatic arthritis would increase by 2% every year on average and the incidence would increase by 2.5% every year from 2019 onwards; Scenario 4: The mortality of psoriatic arthritis would remain constant, and the incidence would increase by 5% every year from 2019 onwards; and Scenario 5: The mortality of psoriatic arthritis would remain constant, and the incidence would decrease by 5% every year from 2019 onwards

Based on the registered data of 65 million, the conservative estimate of overall PsA prevalence in Germany in 2019 was 0.31% (95% confidence interval 0.28–0.36%). Figure 1 presents the projected age-specific prevalences of PsA for men and women in the five scenarios in 2020, 2030, 2040, and 2050. In general, the prevalence trends show a gradual upward development from 2020 to 2050 in all scenarios.

We note that, in 2020, the curves in all five scenarios are quite stable, and the prevalences at all ages are below 1%, contributing to a very low overall disease prevalence. In scenario 1 (the conservative estimate), the peak prevalences of PsA in 2050 are only slightly over 1% for both genders. In contrast, the prevalences in scenario 4 (increasing incidence of 5%) for 60-year-olds have an upward trend, rising from 1% in 2020 to 3% in 2050 for both genders, which is particularly pronounced for women, reaching around 3.5% in 2050. However, scenario 5 (decreasing incidence of 5%) shows prevalence at all ages remaining relatively low from 2020 to 2050 for both genders, with a prevalence of less than 1% in 2050 (Fig. 1).

Furthermore, women contribute generally higher prevalence in all scenarios than men. In 2050, for scenario 4, we observe an early prevalence peak at the age of 60, while in scenario 5, there is a late prevalence peak at age 80. The prevalence peaks of other scenarios lie between the ages of 60 and 80. After their peaks, all the curves show significant downward trends.

### Projected numbers of psoriatic arthritis cases

**Fig. 2:**
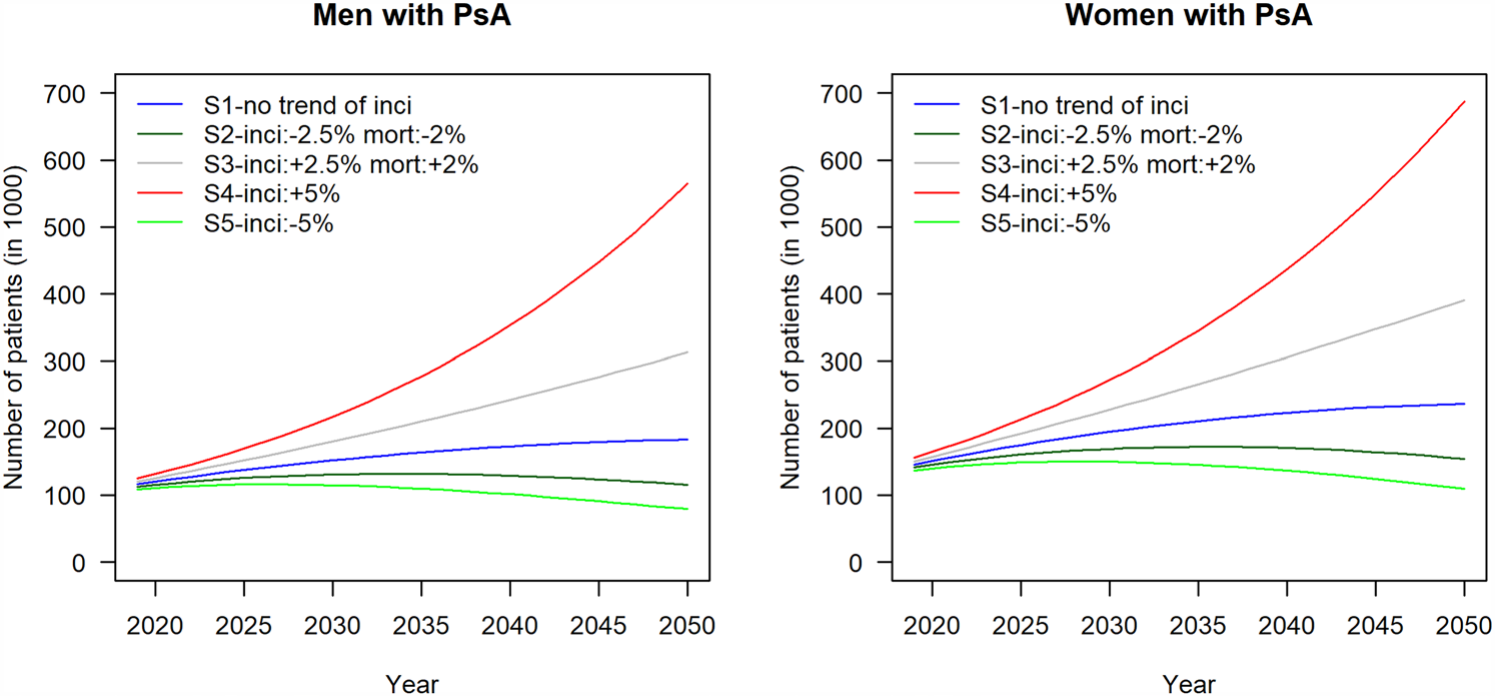
The chronological changes in the projected numbers of cases of psoriatic arthritis for men and women from 2019 to 2050 (in 1000 patients)—Population-Based Projection in Germany. Scenario 1: The incidence and mortality of psoriatic arthritis from 2019 onwards would remain constant and the same as at present; Scenario 2: The mortality of psoriatic arthritis would decrease by 2% every year on average and the incidence would decrease by 2.5% every year from 2019 onwards; Scenario 3: The mortality of psoriatic arthritis would increase by 2% every year on average and the incidence would increase by 2.5% every year from 2019 onwards; Scenario 4: The mortality of psoriatic arthritis would remain constant, and the incidence would increase by 5% every year from 2019 onwards; and Scenario 5: The mortality of psoriatic arthritis would remain constant, and the incidence would decrease by 5% every year from 2019 onwards

Figure 2 shows the chronological changes in the projected numbers of cases of PsA for men and women from 2019 to 2050. For the conservative scenario, cases are projected to increase from 116 thousand in 2019 to 183 thousand in 2050 for men, and from 146 thousand in 2019 to 236 thousand in 2050 for women (Table 2).

**Table 2:** Projected the future numbers of psoriatic arthritis from 2019 to 2050 for both genders in the five different scenarios with the percentage of rate changes—Population-Based Projection in Germany

Scenario	Sex	Projected number (in 1000 population)								Rate changes compared to 2019 (%)			
		2019	2020	2025	2030	2035	2040	2045	2050	2019–2020	2019–2030	2019–2040	2019–2050
S1	Male	116	120	137	152	164	173	179	183	3.4	30.9	49.0	57.8
	Female	146	151	175	194	210	223	232	236	3.6	33.5	53.2	62.2
S2	Male	112	115	126	131	132	129	124	115	2.5	16.4	15.1	2.8
	Female	141	145	160	169	172	170	164	154	2.8	19.8	20.8	9.2
S3	Male	120	125	152	180	210	242	276	313	4.3	49.7	101.5	160.2
	Female	150	157	192	228	265	306	347	391	4.5	51.3	103.2	159.7
S4	Male	125	131	170	217	277	353	448	565	5.4	74.1	183.2	353.2
	Female	156	164	213	272	345	437	550	688	5.5	74.5	180.4	341.6
S5	Male	109	111	116	115	110	101	91	79	1.8	5.4	– 6.9	– 27.4
	Female	137	140	149	150	145	136	124	109	2.1	9.3	– 0.5	– 20.4

The projected numbers of psoriatic arthritis are represented by the chronological changes from 2019 to 2050. These numbers are consistent with the presentations in Fig. 2, with the unit in 1000 population.

The rate change was calculated according to the number differences of the cases of PsA between the specific year and 2019 as numerator, divided by the number of the cases of the year of 2019 as denominator

Scenario 1: The incidence and mortality of psoriatic arthritis from 2019 onwards would remain constant and the same as at present; Scenario 2: The mortality of psoriatic arthritis would decrease by 2% every year on average and the incidence would decrease by 2.5% every year from 2019 onwards; Scenario 3: The mortality of psoriatic arthritis would increase by 2% every year on average and the incidence would increase by 2.5% every year from 2019 onwards; Scenario 4: The mortality of psoriatic arthritis would remain constant, and the incidence would increase by 5% every year from 2019 onwards; and Scenario 5: The mortality of psoriatic arthritis would remain constant, and the incidence would decrease by 5% every year from 2019 onwards

Women contribute more diagnoses of PsA, and the rising trend is especially prominent in scenario 4, corresponding to the prevalence curve (Fig. 1), with cases rising from 156 thousand in 2019 to 688 thousand in 2050. Men’s cases in the same scenario are estimated to increase from 125 to 565 thousand over the same period (Table 2). Furthermore, scenario 3 also shows an increase for women from 150 thousand in 2019 to 391 thousand in 2050, and for men from 120 to 313 thousand.

For both scenario 2 and scenario 5, the peak numbers of cases are forecast to be in 2035, with a subsequent downward trend until 2050. Nevertheless, both curves are relatively stable, with low numbers of cases from 2019 to 2050 compared to the other scenarios. In scenario 2, the number of cases levels off for both men and women, with little fluctuation throughout the forecast period (Fig. 2). In contrast, in scenario 5, following the 2035 peak, the forecast number of cases for women decreases from 145 to 109 thousand by 2050, and from 110 to 79 thousand for men (Table 2).

## Discussion

This is the first study to use the illness–death model to project PsA case numbers in Germany until 2050, accounting for epidemiological parameters based on the National Statutory Health Insurance data of 65 million German population. The novelty of this study is that it not only fills a gap in the rheumatology research in Germany, but also provides PsA reference data for public health stakeholders to aid in decisions on medical resource allocation and pharmaceutical prevention in order to flatten the epidemiological curve and to improve the quality of life of patients with PsA, particularly among the senior female population (Table 3).

**Table 3:** Significance of this Study—Population-Based Projection in Germany

What this study adds
The first study is to use the illness–death model to project PsA prevalence and case numbers in Germany until 2050
The research is to generate assumed population-based data on PsA in Germany that can serve as a reference for public health stakeholders
The German PsA prevalence is similar to that of Denmark, significantly higher than the Czech Republic and North America, and significantly lower than Norway, Spain, and Italy
We would expect worryingly high numbers in the coming decades, if preventive strategies are not implemented
The prevalence curve may flatten and enter a decreasing trend from 2035 to 2050 for both genders, if PsA patient management is put in place, as the World Health Organization advocates
How this study might affect practice or policy
Improved national surveillance of PsA—particularly population-based surveillance data—would be useful for monitoring the trends and development of the disease in Germany
The introduction of effective interventional approaches, such as early diagnosis, active screening, early referral to rheumatology clinics, and appropriate treatment, are excellent tools for flattening the epidemiological curve
At the national level, awareness of clinical realities, such as the shortage of rheumatologists and issues with coding PsA diagnoses, is important, and healthcare stakeholders should ensure that more rheumatologists are trained in order to alleviate the clinical problems experienced by PsA patients

**Fig. 3:**
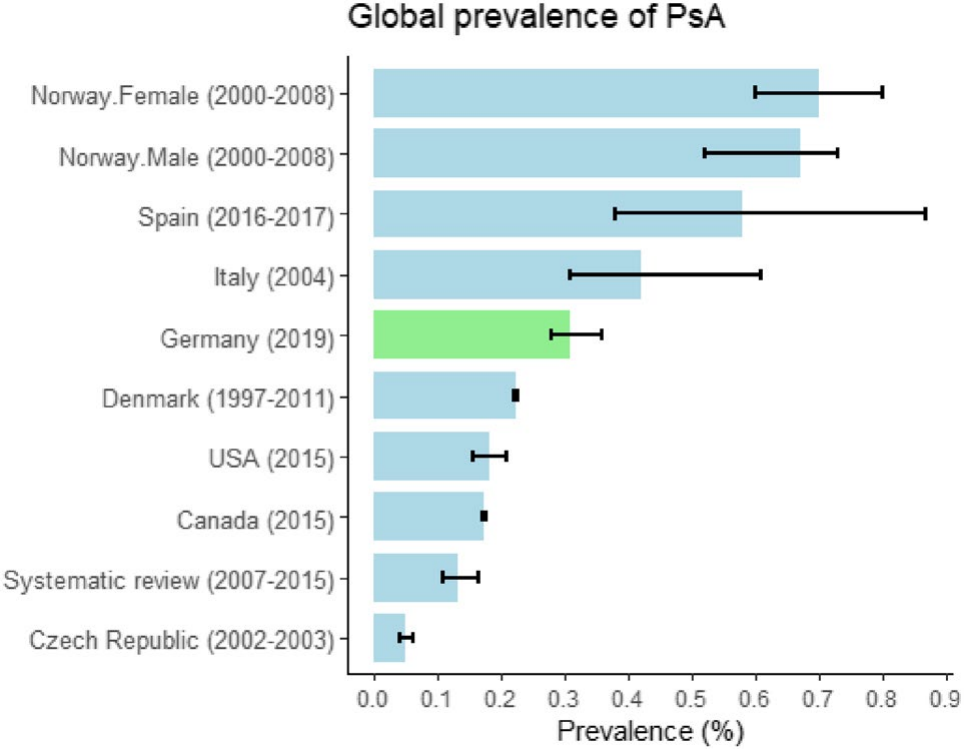
Global prevalence of psoriatic arthritis—Population-Based Projection in Germany. The conservative estimate of overall psoriatic arthritis prevalence in Germany in 2019 was 0.31% (95% confidence interval 0.28–0.36%). The calculation for conservative estimate was considered the total numbers of psoriatic arthritis in scenario 1 in 2019 (as numerator) divided by the total number of German population in 2019 (as denominator) [22]. The data from Canada, Czech Republic, Denmark, Italy, Norway, Spain and the USA were extracted from the rheumatology literature

The conservative estimate of overall PsA prevalence in Germany in 2019 was 0.31%, which is similar to the prevalence in Denmark (0.22% in 1997–2011) [11]; and significantly higher than the prevalence in the USA (0.18% in 2015) [10], Czech Republic (0.049% in 2002–2003) [40], Canada (0.17% in 2015) [13], and in the most recent global systematic review (0.13% in 2007–2015) [7]; and significantly lower than in Norway (0.67–0.70% in 2000–2008) [9], Spain (0.58% in 2016–2017) [41], and Italy (0.42% in 2004) [42]. Figure 3 shows the substantial variations in the global prevalence of PsA.

We found that women have a higher prevalence of PsA, which is consistent with the findings from the Norwegian, Canadian, and Danish studies but inconsistent with the US study [9–11, 13]. Senior women have a higher prevalence than younger women in the scenarios with increasing incidence (Fig. 1), which is also consistent with the Danish study’s finding of PsA being predominant among older women if there is rising incidence [11]. This may be because screening tools are helpful in detecting PsA patients [43, 44], or because older female patients are more likely to seek medical therapy for arthritic symptoms [11]. Another interesting finding is that, in scenario 4 with 5% increasing incidence, even though the number of cases among women (688 thousand) is higher than among men (565 thousand) in 2050, the rate of increase for men between 2019 and 2050 is greater, at 353%, than it is for women, at 341% (Table 2).

In the recent rheumatology literature, there have been few studies describing future projections of the development of PsA prevalence over time, and the current data offer a good reference for decisions regarding PsA patient management. In the conservative scenario (scenario 1), the prevalence of PsA between 2020 and 2050 fluctuates and exceeds 1% only slightly for both genders. This is an encouraging message that shows that maintaining a low disease prevalence until 2050 would represent a possible success in disease prevention efforts. However, in the scenarios with increasing incidence, the peak prevalence for women at the age of 60 may increase markedly, from 1% in 2019 to 3.5% in 2050. In Germany in 2050, an estimated 27% (20 million) of the population will be over 67 years old [22], and the literature indicates that PsA patients are at increased risk for coronary calcification and therefore cardiovascular events and death [45]; the economic burden and mortality from PsA are therefore likely to rise.

From a clinical perspective, a major factor influencing PsA patient management and increased incidence may be the shortage of rheumatologists in Germany. The American College of Rheumatology has estimated that the US demand for patient care services in rheumatology will exceed supply by 4.1 thousand clinical full-time equivalents by 2030 [46], and Germany may face a similar situation, including long waiting lists for specialists that result in delayed treatment and increased incidence of PsA.

Another factor influencing the presented scenarios may be the coding of PsA diagnoses, with most such diagnoses in Germany currently being coded by general practitioners (GP). The coding of a diagnosis (ICD-10) depends firstly on knowledge of the specific diagnosis, which is most commonly held by specialists, and secondly on the availability of therapies: if a GP cannot prescribe a specific PsA medication, the diagnosis is often simply not provided. If patients cannot receive appropriate diagnoses and care from GPs and are not referred to specialists early, this may increase the ultimate incidence. The recent broadening of awareness and knowledge about the disease and its therapeutic options, such as through educational programs or television commercials, could also explain the increase in coded incidence. Such clinical realities could lead to scenarios 3 and 4 and their consequences. It is thus clear that our data could persuade healthcare stakeholders to ensure that more rheumatologists are trained in order to alleviate the clinical problems experienced by PsA patients. Furthermore, the evidence from rheumatology research shows that the endogenous factors of overweight and vitamin D deficiency are likely to increase the incidence of PsA, wherein these possible drivers are increasing, especially in the Western world [47, 48].

The World Health Organization has started advocating for psoriasis patient management [44], but, according to the rheumatology literature, predicting the development of PsA for those already suffering from psoriasis is difficult. Nevertheless, preventive strategies can focus on identifying predictors (e.g., skin and musculoskeletal inflammation, certain comorbidities, and moderate or severe stress) and on treating psoriasis symptoms with individualized therapy strategies early by referring to the specialists [44, 49], and it would thus be possible to slow or even prevent the transition of psoriasis to PsA [49]. With such early interventions to reduce the incidence and to flatten the prevalence curve, like scenarios 2 and 5 in the graph, we might even achieve a decreasing trend in cases from 2035 to 2050, reducing the burden of PsA on the healthcare system (Fig. 2).

The main strengths of this study include its national and population-based data, its large sample size, and the use of the illness–death model in a predictive mathematical estimation of future PsA prevalence and case numbers until 2050. This prospective projection research provides advantages in understanding the trends and developments of PsA over US and Canadian surveys that have examined the prevalence of PsA retrospectively [10, 13]. Our data from 65 million insured data yields a representative and generalizable analysis. However, this study also has limitations. First, the historical data used for the predictions only included patients with public health insurance, thus excluding those with private health insurance or no insurance. However, health insurance is required by law in Germany, so the numbers of those uninsured is very small, and only around 10% are privately insured [20]; thus, although this is a limitation, it is not as profound as it might appear. Second, given that our analysis relies on aggregated insured data, inherent limitations exist due to the absence of detailed documentation regarding the specific diagnostic criteria used for determining PsA [50]. Furthermore, the extrapolated calculation until 2050 relied on insurance claims diagnoses (with possibility of misdiagnosis or overlapping diagnoses) instead of clinically confirmed diagnoses and validations, which may introduce potential bias into the results. Third, we applied the values of the Danish MRR of PsA as an epidemiological parameter into our projection model [12], and as the MRR was used as a pooled value for both sexes, this may represent a disadvantage in the model. Fourth, the five scenarios were developed on the basis of assumptions for the development of disease characteristics, along with the existing literature. Nevertheless, this is the best knowledge available for predicting future situations for PsA. Fifth, our conservative projections are premised on the idea that changes in incidence and mortality will remain constant annually, in accordance with historical trends in disease development. Consequently, our model and calculations would struggle to predict the effects of (i) the abrupt emergence of COVID-19 and (ii) specific time-bound treatment campaigns by pharmaceutical companies or political changes in health policy.

In conclusion, this study fills a gap in the research on the epidemiological burden and future development of PsA until 2050. We observed that the conservative estimate of overall PsA prevalence in Germany in 2019 was 0.31% and that, until 2050, senior women are projected to have a higher prevalence of 3.5% and to be more vulnerable to PsA. Our models aim to convince public health stakeholders to understand the shortcomings and weaknesses of current PsA patient management and to appreciate that, in the long term, it will be necessary to implement preventive strategies to identify predictors and treat psoriasis symptoms early in order to delay or even prevent the transition of psoriasis to PsA.

## Supplementary Information

Below is the link to the electronic supplementary material.

Supplementary file1 (DOCX 73 KB)

## Data Availability

Due to the data protection laws in Germany, the datasets generated and/or analyzed during the current study cannot be made publicly available. The German law prohibits that the claims data affecting about 65 million people are used for other purposes than for research. Access to the data are granted to qualified research institutions upon request at the Forschungdatenzentrum (FDZ) in accordance with §§ 303a, 303f of Sozialgesetzbuch (SGB) V. Detailed information regarding the process for applying to obtain data access can be found on the website of the FDZ (in German) (https://www.forschungsdatenzentrum-gesundheit.de/das-fdz). Additionally, relevant laws pertaining to this matter are available on the website (in German) (https://www.gesetze-im-internet.de/sgb_5/BJNR024820988.html#BJNR024820988BJNG008700308).

## Abbreviations

DIMDI: German institute for medical documentation and information
GP: General practitioners
ICD-10: The 10th edition of the international statistical classification of diseases and related health problems
MRR: Mortality rate ratio
PsA: Psoriatic arthritis
