# Supplementary material for: A population-based projection of psoriatic arthritis in Germany until 2050: analysis of national statutory health insurance data of 65 million German population
Source: Rheumatol Int. 2023 Aug 19;43(11):2037–47. doi: 10.1007/s00296-023-05422-2 (PMC10495508; doi:10.1007/s00296-023-05422-2)
Supplement: Supplementary file 1 — Supplementary file1 (DOCX 73 KB) [file 296_2023_5422_MOESM1_ESM.docx]

**Title: A Population-Based Projection of Psoriatic Arthritis in Germany until 2050: Analysis of National Statutory Health Insurance Data of 65 Million German Population**

**Supplementary materials**

- **Supplementary Table 1 Cubic Spline Interpolation**
- **Supplementary Table 2A Summary of Psoriatic Arthritis Incidence Changes in Large Population-Based Studies**
- **Supplementary Table 2B Summary of Psoriatic Arthritis Mortality Changes in Large Population-Based Studies**
- **Supplementary Figure 1 The illness-death model relevant to our study**

**Supplementary Table 1 Cubic spline interpolation**

|  | **Cubic spline** | **R codes** | **Interpretation of the codes** |
| --- | --- | --- | --- |
| **Prevalence** | The use of a cubic spline in a regression model like ours allows for the possibility that the relationship between the age variable and the logit-transformed prevalence of psoriatic arthritis is not simply linear but may exhibit more complex, non-linear patterns. By fitting a spline, the model can potentially capture these patterns more accurately | mod_lp <- lm(logit(prev) ~ t*m*ns(a, df = 3), data = prev) | “lm” represents the linear model function, “prev” refers to the historical prevalence, “t” stands for the year, “m” indicates the gender, “a” symbolizes the age group, and “df = 3” defines the degrees of freedom |

**Note: A cubic spline is a piecewise cubic function that smoothes a given set of data points [1].**

**Supplementary Table 2A Summary of Psoriatic Arthritis Incidence Changes in Large Population-Based Studies -Population-Based Projection in Germany**

| **Scenario** | **Authors, Years, Countries** | **Data extraction** | **Population-based** | **N events of PsA** | **Person-years** | **Incidence Rate** | **Changes of IR** |
| --- | --- | --- | --- | --- | --- | --- | --- |
| Scenario 2 | Guldberg-Møller *et al*, 2019, Denmark [2] | 2005-2008 | Nationwide register-based data | 12,128 | 115,198 | 0.02176 | ≈ 2.5% (↓) |
|  | Guldberg-Møller *et al*, 2019, Denmark [2] | 2009-2012 | Nationwide register-based data | 316 | 14,921 | 0.02117 |  |
| Scenario 3 | Love *et al*, 2012, UK [3] | 1995-2010 | Claim data | 976 | 368,302 | 0.00264 | ≈ 2.5% (↑) |
|  | Green *et al*, 2019, UK [4] | 1998-2014 | Claim data | 1,409 | 521,826 | 0.00270 |  |
| Scenario 4 | Karmacharya *et al*, 2021, USA [5] | 1970–1999 | Data resources of the Rochester Epidemiology Project (REP) | - Between 1970 and 1999, the incidence of PsA increased at a rate of 4% per year (RR=1.04 [95% CI: 1.02–1.06]) | | | ≈ 5.0% (↑) |
|  | Karmacharya *et al*, 2021, USA [5] | 2000–2017 | Data resources of the Rochester Epidemiology Project (REP) | - In 2000-2017, there is no evidence of an increase in PsA incidence overall - In 2000-2017, there was a 3% annual increase in women (RR=1.03 [95% CI: 0.99–1.08]) | | |  |
| Scenario 4 | Eder *et al*, 2019, Canada [6] | 2008 | Health administrative databases from Ontario, Canada | 1,386 | 9,829,603 | 0.000141 (Crude incidence) | ≈ 5.0% (↑) |
|  | Eder *et al*, 2019, Canada [6] | 2015 | Health administrative databases from Ontario, Canada | 1,647 | 10,774,802 | 0.000152 (Crude incidence) |  |
| Scenario 4 | Iskandar *et al*, 2022, Taiwan [7] | 2009 | Taiwan National Health Insurance database | N/A | N/A | 0.00062 (PsO) | ≈ 5.0% (↑) |
|  | Iskandar *et al*, 2022, Taiwan [7] | 2017 | Taiwan National Health Insurance database | N/A | N/A | 0.00065 (PsO) |  |
| Scenario 5 | Hoff *et al*, 2015, Norway [8] | 2000–2008 | Data from the Nord-Trøndelag Health Study 3 (HUNT 3) | - In the 9-year period of 2000–2008, a total of 188 patients were diagnosed with PsA, which give an incidence rate of 41.3 per 100,000 (35.8–47.6) | | | ≈ 5.0% (↓) |
|  | Kerola *et al*, 2021, Norway [9] | 2011-2015 | Data from the Norwegian Patient Registry (NPR) | - PsA incidence per 100,000 PY was 25.9 (95% CI 25.2-26.6), when using the base case definition, 29.0 (95% CI 28.2-29.7) for the broad definition | | |  |

**Note: IR: Incidence rate; PsA: Psoriatic Arthritis; PY: Person-years; RR: Risk Ratio;**

**Note: N/A: Not Applicable;**

**Supplementary Table 2B Summary of Psoriatic Arthritis Mortality Changes in Large Population-Based Studies - Population-Based Projection in Germany**

| **Scenario** | **Authors, Years, Countries** | **Data extraction** | **Population-Based** | **Mortality** | **Mortality Changes** |
| --- | --- | --- | --- | --- | --- |
| Scenario 2 | Springate et al, 2017, UK [10] | 1999-2013 | UK Clinical Practice Research Datalink (CPRD) | - There was an overall reduction in mortality over time for all patients with and without psoriasis over the 15-year study period (HR 0.92 per year, 95% CI 0.91–0.92) - Mortality among patients diagnosed with psoriasis at an older age did not appear to be increased (age 80+ years: HR 0.99, 95% CI 0.91–1.08) | ≈ 2.0% (↓) |
| Scenario 2 | Iskandar et al, 2022, Taiwan [7] | 2009-2017 | Taiwan National Health Insurance database | - Overall reduction in all-cause mortality over time for all patients with psoriasis and psoriatic arthritis was reported (HR 0.99, 95% CI 0.98–0.99) | ≈ 2.0% (↓) |
| Scenario 2 | Elalouf et al, 2020, Canada [11] | 1978-2017 | Patients enrolled into the University of Toronto PsA clinic | - The overall standardized mortality ratios (SMR) of the PsA comparing the mortality to the general population of Ontario between 1978 and 2017 was 0.92 (95% CI: 0.80-1.05) - For the age groups of 40-59, SMR was 0.97 (95% CI: 0.68-1.34) | ≈ 2.0% (↓) |
| Scenario 3 | Kerola et al, 2022, Norway [9] | 2010-2017 | Data from the Norwegian nationwide  register-linkage study | - Men with PsA had a slightly increased mortality rate ratio with 1.02 [95% CI: 0.93-1.11] | ≈ 2.0% (↑) |
| Scenario 3 | Chaudhary et al, 2021, Systematic review [12] | N/A | Data from the systematic review | - Men with PsA had a higher all-cause mortality (RR 1.02 [95% CI 0.66–1.59]) | ≈ 2.0% (↑) |

**Note: HR: Hazard ratio; PsA: Psoriatic Arthritis; RR: Rate ratio; SMR: Standardized mortality ratios**

**Supplementary Figure 1 The illness-death model relevant to our study**

*Note: The illness-death model*

**Stage 2**

**(Psoriatic arthritis)**

**Stage 1**

**(No psoriatic arthritis)**

**Stage 3**

**(Death)**

***IR(t,a)***

***m_1_(t,a)***

***m_0_(t,a)***

Note: This figure provides a summary of the technical detail of the illness-death model. The arrows indicate transition rates between states. These transition rates are dependent on the calendar time *t* and age *a*. The function *IR(t,a)* represents the incidence rate from the state of “No psoriatic arthritis” to “Psoriatic arthritis”, depending on calendar time *t* and age *a*. Similarly, *m_0_(t,a)* and *m_1_(t,a)* are the mortality rates among people without and with psoriatic arthritis, respectively [13, 14].

**References**

**1.** McClarren RG (2018) Chapter 10 - Interpolation. In: McClarren RG (ed) Computational Nuclear Engineering and Radiological Science Using Python, Academic Press, pp 173-192. https://doi.org/10.1016/B978-0-12-812253-2.00012-1

**2.** Guldberg-Møller J, Cordtz RL, Kristensen LE et al (2019) Incidence and time trends of joint surgery in patients with psoriatic arthritis: a register-based time series and cohort study from Denmark. Ann Rheum Dis 78(11):1517-1523. <https://doi.org/10.1136/annrheumdis-2019-215313>

**3.** Love TJ, Zhu Y, Zhang Y et al (2012) Obesity and the risk of psoriatic arthritis: a population-based study. Ann Rheum Dis 71(8):1273-1277. <https://doi.org/10.1136/annrheumdis-2012-201299>

**4.** Green A, Shaddick G, Charlton R et al (2020) Modifiable risk factors and the development of psoriatic arthritis in people with psoriasis. British Journal of Dermatology 182(3):714-720. <https://doi.org/10.1111/bjd.18227>

**5.** Karmacharya P, Crowson CS, Bekele D et al (2021) The Epidemiology of Psoriatic Arthritis Over Five Decades: A Population-Based Study. Arthritis Rheumatol 73(10):1878-1885. <https://doi.org/10.1002/art.41741>

**6.** Eder L, Widdifield J, Rosen CF et al (2019) Trends in the Prevalence and Incidence of Psoriasis and Psoriatic Arthritis in Ontario, Canada: A Population-Based Study. Arthritis Care Res (Hoboken) 71(8):1084-1091. <https://doi.org/10.1002/acr.23743>

**7.** Iskandar IYK, Chen TC, Chen LC et al (2022) Incidence, Prevalence, and Mortality of People with Psoriasis and Psoriatic Arthritis in Taiwan: A Nationwide Cohort Study. Acta Derm Venereol 102:adv00807. <https://doi.org/10.2340/actadv.v102.1962>

**8.** Hoff M, Gulati AM, Romundstad PR et al (2015) Prevalence and incidence rates of psoriatic arthritis in central Norway: data from the Nord-Trøndelag health study (HUNT). Ann Rheum Dis 74(1):60-64. <https://doi.org/10.1136/annrheumdis-2013-203862>

**9.** Kerola AM, Sexton J, Wibetoe G et al (2021) Incidence, sociodemographic factors and treatment penetration of rheumatoid arthritis and psoriatic arthritis in Norway. Semin Arthritis Rheum 51(5):1081-1088. <https://doi.org/10.1016/j.semarthrit.2021.08.006>

**10.** Springate DA, Parisi R, Kontopantelis E et al (2017) Incidence, prevalence and mortality of patients with psoriasis: a U.K. population-based cohort study. Br J Dermatol 176(3):650-658. <https://doi.org/10.1111/bjd.15021>

**11.** Elalouf O, Muntyanu A, Polachek A et al (2020) Mortality in psoriatic arthritis: Risk, causes of death, predictors for death. Semin Arthritis Rheum 50(4):571-575. <https://doi.org/10.1016/j.semarthrit.2020.04.001>

**12.** Chaudhary H, Bohra N, Syed K et al (2021) All-Cause and Cause-Specific Mortality in Psoriatic Arthritis and Ankylosing Spondylitis: A Systematic Review and Meta-Analysis. Arthritis Care Res (Hoboken). <https://doi.org/10.1002/acr.24820>

**13.** Putter H, Fiocco M, Geskus RB (2007) Tutorial in biostatistics: competing risks and multi-state models. Stat Med 26(11):2389-2430. <https://doi.org/10.1002/sim.2712>

**14.** Tönnies T, Röckl S, Hoyer A et al (2019) Projected number of people with diagnosed Type 2 diabetes in Germany in 2040. Diabet Med 36(10):1217-1225. <https://doi.org/10.1111/dme.13902>
